# Combined thoracoabdomial injury: Case report

**DOI:** 10.1016/j.amsu.2020.06.030

**Published:** 2020-06-26

**Authors:** A. Muratov, Z. Tuibayev, Z. Arynov, K. Abdykalykov, O. Kurbanbayev, B. Khashimov, M. Matkasymov, Z. Abdullaeva

**Affiliations:** a Osh State University, Department of General Surgery, Kurmanjan Datka Street 157, 723500, Osh, Kyrgyzstan; b Osh City Clinical Hospital, Surgery Department, Kurmanjan Datka Street 157, 723500, Osh, Kyrgyzstan; c Osh State University, International Medical Faculty, Department of Surgical Disciplines and Traumatology, Jolon Mamytov Campus, 723500, Osh, Kyrgyzstan; d Jalal-Abad Regional Hospital, Department of Surgery, Pushkin Str. 92, 715609, Jalal-Abad, Kyrgyzstan; e Tumen Regional Clinical Hospital, Department of Thoracoabdominal Surgery, Russian Federation; f Department of Thoracoabdominal Surgery, Osh Interregional Clinical Hospital, Osh, Kyrgyzstan; g Osh State University, International Medical Faculty, Department of Anatomy, Histology and Normal Physiology, Jolon Mamytov Campus, 723500, Osh, Kyrgyzstan

**Keywords:** Thoracoabdominal injuries, Trauma, Thorax, Bleeding, Case report, Emergency

## Abstract

**Introduction:**

Combined thoracoabdominal injuries are the most severe case in the medical practice. Bilateral injuries are rare case in patients and just few cases reported in literature. Diaphragm traumas and pneumothorax are defined as severe trauma leading to injuries of thoracic and abdominal organs. We are describing multiple injuries of abdominal tract and chest causing internal bleeding and hemothorax in patient. Surgical operation was carried out on the right side thoracotomy including revision of the pleural cavity and subphrenic space, closure of the wound, removal of the blood clot and drainage of the pleural with abdominal cavities.

**Presentation of case:**

A 24-years old man was admitted in Emergency Department after penetrating knife wounds of abdominal cavity.

**Discussion:**

X-ray of the tracheobronchial tree (bronchography) showed closed chest injuries, post traumatic hemopneumothorax of the right side.

**Conclusion:**

Surgical repair was conducted by displacing of the organs in chest cavity with lamellar T-shaped retractor, and hemostatic Billroth, Kocher clamps to stop bleeding in the wound.

## Highlights

• Diaphragm traumas and pneumothorax are defined as severe trauma leading to injuries of thoracic and abdominal organs.
• Along hemostasis, pleural cavity containing 200 ml clot and 300 ml liquid blood was opened by electrocoagulation method.
• Patient lungs wounds were sutured from pleural cavity with nodular and double-row sutures.
• After control for hemostasis, layered sutures on the wound and aseptics were applied.

## 1 Introduction

Combined organ injuries, including thoracoabdominal injuries, constitute the most severe type of injury that is unpredictable in terms of consequences, accompanied by high mortality comparing to injuries inside a single cavity; it was reported that patients with penetrating thoracoabdominal injuries have a 20–30% risk of other complicated injuries [[1], [2], [3], [4]]. Activating the identification process of subsequent adoption of optimal, tactical and technical solutions for surgical interventions are important in the determination of wounds nature and their prognosis [5,6]. Hemothorax after blunt trauma is a rare clinical occurrence and associated with significant morbidity and mortality cases [7]. According to the current standards, low morbidity and mortality in the trauma patients may be achieved by a multidisciplinary and experienced trauma team [8].

## 2 Case report

**Fig. 1:**
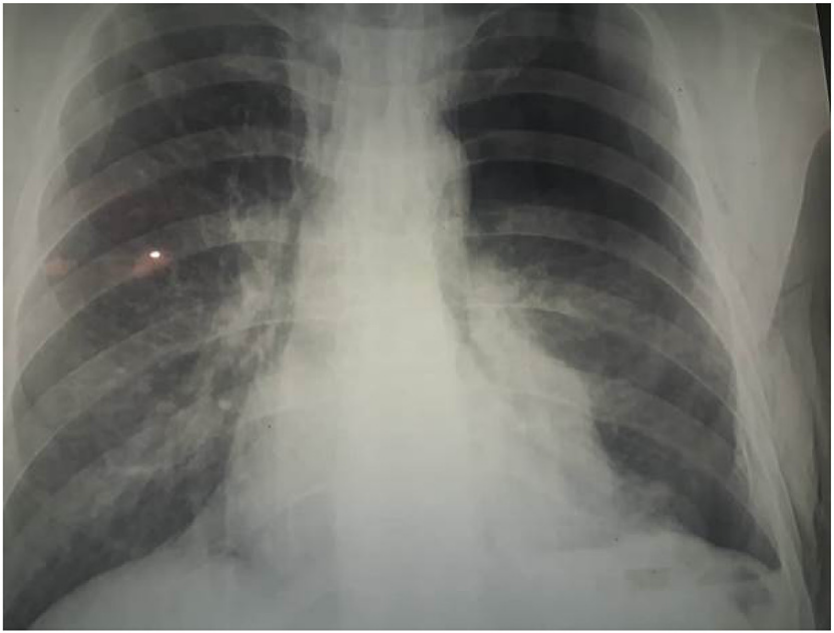
Chest X-ray tomography detection of hemopneumothorax on the right side.

A 24-years old man was admitted in Emergency Department after penetrating knife wounds of abdominal cavity. Chest X-ray tomography detected pneumothorax on the right side (Fig. 1), pneumoperitoneum and thoracic-abdominal effusion with internal organ injuries. Degree of the diaphragm injury was determined as degree III according to the American association for the surgery of trauma organ injury scale for diaphragmatic injuries (Table 1) [9].

**Table 1:** American association for the surgery of trauma organ injury scale for diaphragmatic injuries (reproduced with permission of Springer from DeBarros & Martin, 2015).

Grade^a^	Description of injury
I	Contusion or hematoma without rupture
II	Laceration <2 cm
III	Laceration 2–10 cm
IV	Laceration >10 cm with tissue loss ≤25 cm^2^
V	Laceration with tissue loss >25 cm^2^

a Advance one grade for bilateral injuries up to grade III.

## 3 Surgical techniques

**Fig. 2:**
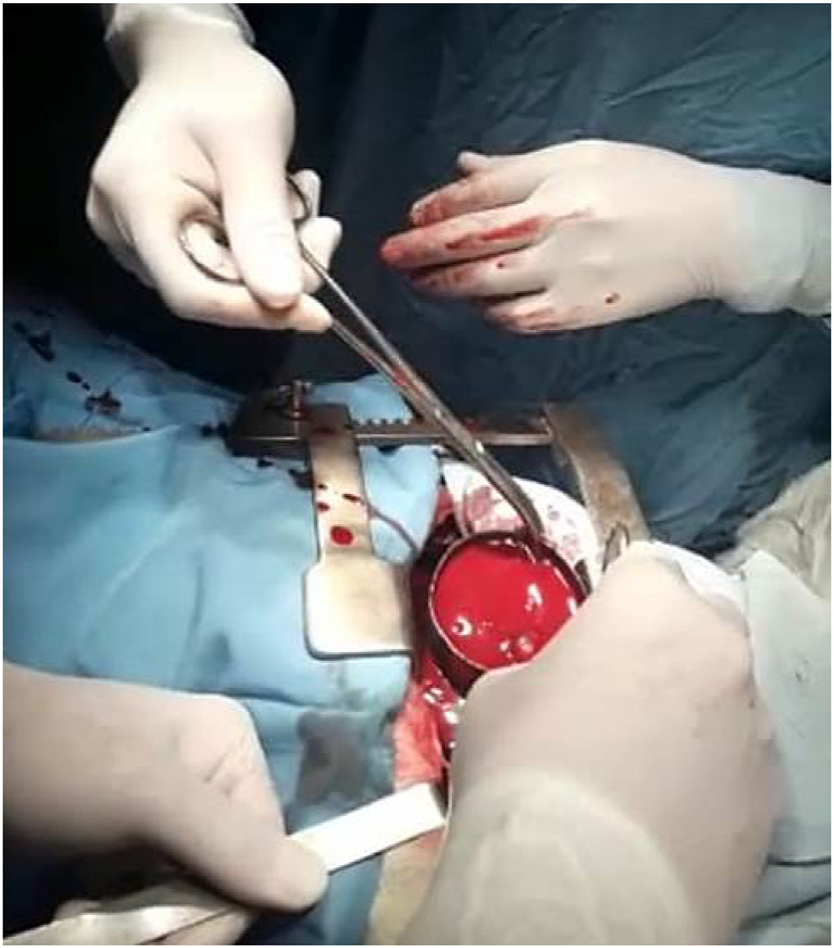
Clot removing from the pleural cavity.

**Fig. 3:**
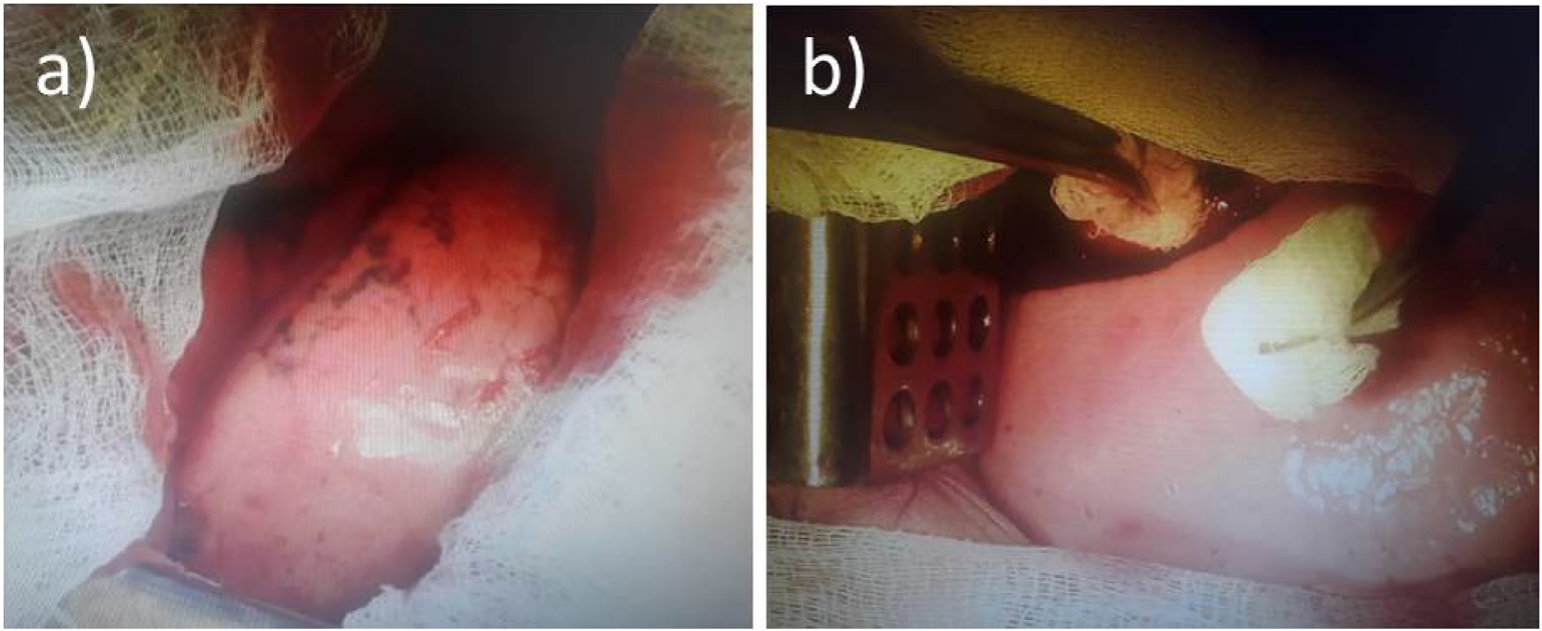
a) Sanitizing patients' lungs and pleura; b) removing of the blood clot during hemothorax.

**Fig. 4:**
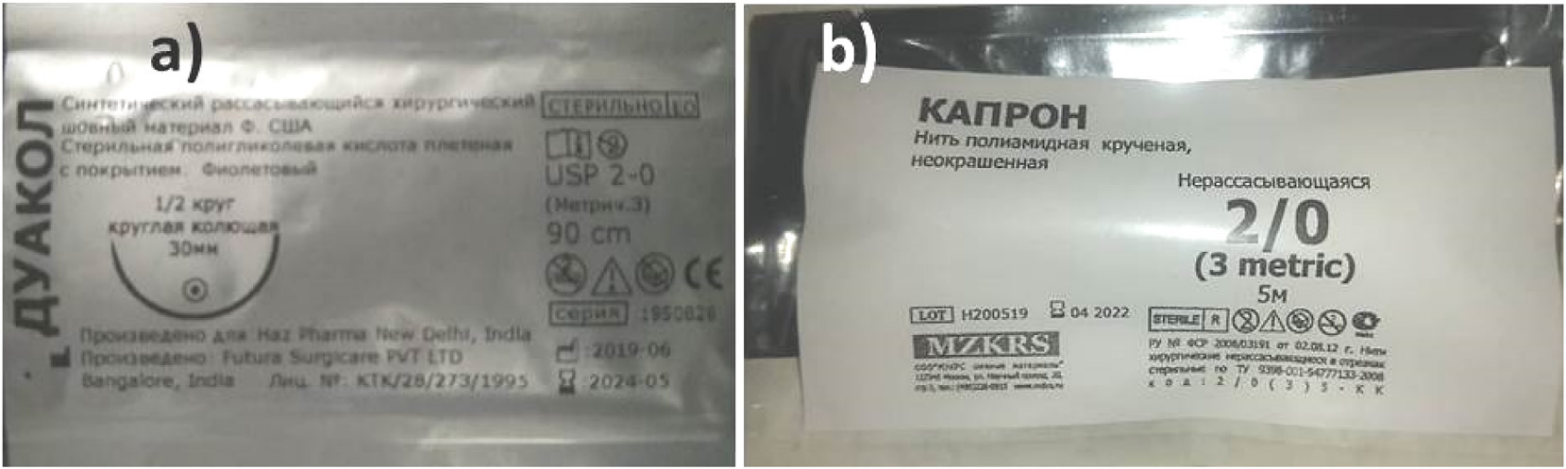
a) Non-absorbable suture Duakol (India) with atraumatic needles used for repair of diaphragm wounds; b) Capron polyamide suture (Russia) used for patients lung wounds repair.

**Fig. 5:**
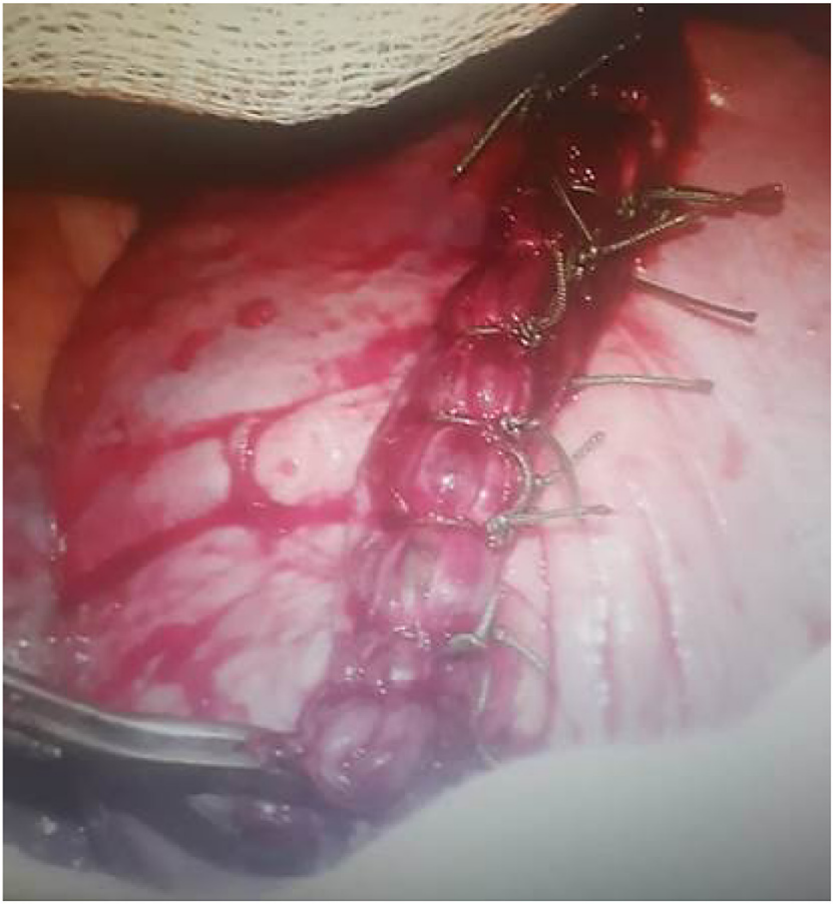
Patients lung wounds were repaired by the nodular and double-row sutures.

Our tactic in choosing the priority of surgical interventions had a number of fundamental advantages. Surgical interventions were performed by group of surgeons: after disinfection of an operative table, incision along the VII intercostal space was made by an anterolateral access. Along the hemostasis, pleural cavity containing 200 ml of a clot and 300 ml of liquid blood was opened by electrocoagulation method [10,11]. After blood clot removing (Fig. 2), pleural cavity was lavaged with electric suction pump and rinsed. A linear wound was found on the diaphragm with the length of 2 cm, where pleural cavity was linked with the subphrenic space. For inspection of the abdominal cavity the wound on the diaphragm was enlarged, and damage of a capsule on the liver was detected, which was determined as 1 cm in length, and the blood clots in subphrenic space were removed. Lungs were lavaged and hold by specialty retractors [12] to displace the bones of the shoulder (Fig. 3). Main repair was performed with non-absorbable sutures (Fig. 4) violet color Duakol (Futura Surgicare PVT, India) 90 cm with atraumatic needles 1/2 round, 30 mm, and Capron polyamide sutures 2/0(3)5-KK (Russia). The patients lung and diaphragm wounds were sutured from the pleural cavity, nodular and double-row sutures were applied (Fig. 5).

## 4 Discussion

No blood was found around the liver and abdominal cavity. The wound on the liver capsule was not bleeding; it was coagulated, after consultation by an abdominal surgeon. Liver tissue was not damaged. Subphrenic space and abdominal cavity were drained. Double-row sutures were placed on the wounds of the diaphragm.

The pleural cavity was drained. Damage of the pleura along the penetrating wound was sutured. After control for hemostasis, layered sutures on the wound and aseptics were applied. Our clinical observations showed that, operation tactics during thoracotomy with right-sided thoracoabdominal wounds depend on the size of the liver wound, during the left-sided thoracoabdominal wounds an operation tactic depends on the location of the diaphragm wound [13].

Post operation diagnosis was penetrating thoracoabdominal wound on the right side. Right lung, diaphragm and damage of the liver capsule, right side hematopneumatic and posthemorrhagic shock were observed.

## Disclaimer

The paper has been reported in line with the SCARE criteria [14]. This research has received no external funding. The authors declare no conflict of interest.

## Ethical approval

Procedures were followed in accordance with the ethical standards of the Helsinki Declaration (1964, amended most recently in 2008) of the World Medical Association.

## Consent

Written consent was obtained for publishing of this case report.

## Author contributions

Abdizhalil Muratov, Zair Tuibayev and Zamir Arynov: Conceptualization, Methodology, Data curation, Writing-Original draft preparation. Kozubai Abdykalykov, Omurbek Kurbanbayev, B. Khashimov and Marat Matkasymov: Contributed in Designing of this Study. Zhypargul Abdullaeva: Writing-Reviewing, Editing and Submission.

## Provenance and peer review

Not commissioned, externally peer reviewed.

## Funding

This work received Government Funding No. 44071031030033026.

## Data availability

Abdullaeva, Zhypargul (2020), “Case report”, Mendeley Data, V2, https://doi.org/10.17632/h3dhbkfwtv.2.

## Declaration of competing interest

The authors declare no conflict of interest.
